# Amyloidosis: What does pathology offer? The evolving field of tissue biopsy

**DOI:** 10.3389/fcvm.2022.1081098

**Published:** 2022-12-05

**Authors:** Mattia Riefolo, Matteo Conti, Simone Longhi, Benedetta Fabbrizio, Ornella Leone

**Affiliations:** ^1^Cardiovascular and Cardiac Transplant Pathology Unit, IRCCS Azienda Ospedaliero-Universitaria di Bologna, Bologna, Italy; ^2^Department of Experimental, Diagnostic and Specialty Medicine, University of Bologna, Bologna, Italy; ^3^Pharmacology Unit, IRCCS Azienda Ospedaliero-Universitaria di Bologna, Bologna, Italy; ^4^Public Health Department, AUSL Imola, Bologna, Italy; ^5^Department of Cardiology, IRCCS Azienda Ospedaliero-Universitaria di Bologna, Bologna, Italy; ^6^Department of Pathology, IRCCS Azienda Ospedaliero-Universitaria di Bologna, Bologna, Italy

**Keywords:** cardiac amyloidosis (CA), pathology, diagnosis, biopsy, proteomics

## Abstract

Since the mid-nineteenth century pathology has followed the convoluted story of amyloidosis, recognized its morphology in tissues and made identification possible using specific staining. Since then, pathology studies have made a significant contribution and advanced knowledge of the disease, so providing valuable information on the pathophysiology of amyloid aggregation and opening the way to clinical studies and non-invasive diagnostic techniques. As amyloidosis is a heterogeneous disease with various organ and tissue deposition patterns, histology evaluation, far from offering a simple yes/no indication of amyloid presence, can provide a wide spectrum of qualitative and quantitative information related to and changing with the etiology of the disease, the comorbidities and the clinical characteristics of patients. With the exception of cardiac transthyretin related amyloidosis cases, which today can be diagnosed using non-biopsy algorithms when stringent clinical criteria are met, tissue biopsy is still an essential tool for a definitive diagnosis in doubtful cases and also to define etiology by typing amyloid fibrils. This review describes the histologic approach to amyloidosis today and the current role of tissue screening biopsy or targeted organ biopsy protocols in the light of present diagnostic algorithms and various clinical situations, with particular focus on endomyocardial and renal biopsies. Special attention is given to techniques for typing amyloid fibril proteins, necessary for the new therapies available today for cardiac transthyretin related amyloidosis and to avoid patients receiving inappropriate chemotherapy in presence of plasma cell dyscrasia unrelated to amyloidosis. As the disease is still burdened with high mortality, the role of tissue biopsy in early diagnosis to assure prompt treatment is also mentioned.

## Introduction

Biopsy is the most reliable method to show up amyloid fibrillar deposits within organs and tissues. These deposits derive from a variety of abnormally aggregated precursor proteins, and can cause cytotoxicity-mediated lesions, distortion of tissue architecture and organ dysfunction. The term amyloidosis indicates the disease that can result from pathologic protein aggregation and includes a wide range of systemic or localized disorders, highly heterogeneous in terms of cause, clinical manifestation, anatomic distribution, progression, and prognosis (1).

Although the first mentions of amyloidosis in the spleen date back to autopsy reports of the seventeenth century, the term “amyloid” was introduced in medical literature and popularized in 1854 by the German pathologist Rudolph Virchow (2, 3). He used this term for a substance found in human tissue, similar to that previously described at autopsy as “stony,” “gelatinous,” “lardaceous,” or “waxy” visceral material. Virchow most likely considered amyloid to be similar to starch, a kind of animal cellulose, although at that time the distinction between starch and cellulose was unclear. In 1859 the German chemist August Kekule presumed that the material infiltrating organs was mainly constituted by “albumoid compounds,” but, nonetheless, the name “amyloid” did not change and the use of a unified nomenclature opened the way to biological and clinical research through multidisciplinary collaboration between pathologists, chemists, physicists, and clinical researchers, collaboration still active today (3, 4).

From the turn of the century there were major advances in amyloid studies with the ever more common use of light microscopy, which identified its amorphous structure, and the use of histopathologic dyes such as Congo red (CR) and thioflavin. CR was found to bind avidly to amyloid (5) and to show apple-green birefringence when viewed under polarized light (6, 7).

More detailed submicroscopic and physical studies in the second half of 20th century demonstrated the fibrillary ultrastructure of amyloid (8) and that fibrils were composed of polypeptide chains with generic cross-beta conformation (9, 10). Amino acid sequence analyses gradually led to the discovery that each type of amyloid consists of a different fibril protein and, in the 2000s, the term “amylome” was introduced to describe the multitude of proteins potentially able to generate amyloid-like fibrils, many of which, however, do so only under certain *in vitro* conditions. It is therefore mandatory to clarify exactly what we mean by the term amyloid (11, 12).

To date 40 proteins have been identified in humans as amyloidogenic, 18 of which associated with systemic amyloidosis and 22 with localized disease (1).

In order to achieve a proper classification of amyloidosis, the current goals for clinical management are first to identify amyloid deposits in tissue, then to understand fibril distribution and the anatomical structures involved and, most importantly, to determine protein composition, i.e., to type amyloid.

Following the pioneering methods published around a decade ago, a mass spectrometry-based proteomic approach to amyloid typing revolutionized diagnostic protocols and placed renewed value on information obtained from histology and immunohistochemistry.

The review describes the histologic approach to amyloidosis today, and the current role of tissue screening or targeted organ biopsy protocols in the light of present diagnostic algorithms and various clinical situations, with particular focus on histopathologic patterns in endomyocardial and renal biopsies. Special attention is given to techniques for typing amyloid fibril proteins, necessary for the new therapies available today for cardiac transthyretin amyloidosis and to avoid patients receiving inappropriate chemotherapy in presence of plasma cell dyscrasia unrelated to amyloidosis. As the disease is still burdened with high mortality, the role of early diagnosis using tissue biopsy to assure prompt treatment is also mentioned.

## Pathology examination of amyloidosis

Anatomo-pathological study of organs and tissues is essential in a complex disease like amyloidosis, characterized by a wide spectrum of acquired and hereditary etiologies, various pathogenetic mechanisms, involvement of many organs and tissues and considerable phenotypic heterogeneity (13) (Table 1). Pathology investigation involves various steps, each of which can provide major diagnostic, therapeutic, and prognostic information. Gross and histology examination are performed as well as identification of the precursor proteins in the tissue samples, using various typing methods, ranging from immune-biochemical techniques to molecular proteomic analysis.

**TABLE 1:** Some characteristics of main types of amyloidosis.

Type	Underlying pathologic conditions
AL	Monoclonal protein-secreting disorders (usually clonal plasma cell).
AA	• Associated to long-standing inflammatory process: chronic infections, rheumatological/autoimmune/inflammatory disorders, hereditary auto-inflammatory disease. • Benign tumors. • Various hematological and solid cancers. • Unknown etiology.
ATTRv, AGel AApoAI, AApoAII, AApoCII, AApoCIII, AFib, ALys	Hereditary amyloidoses due to mutations of various gene proteins.
ATTRwt	Aging-related amyloidosis
Aβ2M derived from β2 microglobulin, associated to long-term dialysis. Amyloid deposits derived from insulin and injection of enfuvirtide.	Iatrogenic amyloidosis
Organ-specific amyloidosis	Localized amyloid deposits derived from hormones or local protein precursors of endocrine organs or tumors (e.g., thyroid medullary carcinoma; isolated atrial amyloidosis).

**Main pathogenetic mechanisms**	

• Excess protein production favoring abnormal folding of proteins and their aggregation into insoluble aggregates. • Mutated protein with a higher tendency to misfold. • Intrinsic propensity of normal wild-type protein to misfold and form amyloid fibrils. • Proteolytic remodeling of a wild-type protein into an amyloidogenic fragment.	

Histology is particularly crucial for various reasons (14–16):

1. making a definite diagnosis in cases of clinically unexpected amyloidosis or in dubious cases which require a broader differential diagnosis for organ diseases, something still not infrequent, especially in spoke Hospitals;
2. determining organ and tissue involvement in order to define the systemic or localized nature of the disease;
3. describing type of distribution and anatomical structures involved in single organs;
4. defining organ disease burden;
5. indicating the most pertinent sample for optimal amyloid fibril typing.

According to the 2020 recommendations of the International Society of Amyloidosis nomenclature committee, which state that “in medical practice amyloid is recognized microscopically by its amorphous structure, affinity for the dye Congo red and its increased birefringence under polarized light after such staining,” the cornerstone for diagnosis still rests on identification in tissues of amyloid deposits with their typical microscopic structure and histochemical properties (1).

The following sections describe the standardized step-by step approach that the pathologist should follow to reach a diagnosis of amyloidosis and to provide as much information as possible when examining tissues in this context. For a correct approach the pathologist should have a thorough knowledge of the technical aspects and staining protocols, should be aware of the characteristics of specific tissues when analyzing amyloid deposits and should be properly trained in amyloid morphological findings and in the other alterations and diseases to be considered for a differential diagnosis.

### Main technical aspects to bear in mind when examining amyloid deposits in tissues

The usual formalin-fixed and paraffin-embedded (FFPE) specimens can be used for complete examination and characterization of tissue amyloid deposits. These samples are suitable not only for optimal histological, histochemical, and immunohistochemical investigation, but are also for molecular analysis, as Mayo Clinic proteomic researchers showed (17). Frozen tissue is needed only in those Centers which traditionally use immunofluorescence for amyloid typing.

Tissue fragments fixed in glutaraldehyde solutions are required for ultrastructural examination, although for centers which make use of immunoelectron microscopy for amyloid protein typing this fixative is not always adequate.

Targeted organ biopsies (heart, kidney, liver, bone marrow) are prepared and sectioned according to respective guide-line or consensus document protocols, which already cover serial or multiple sections and unstained slides for further investigation (18–20). Screening biopsies (labial salivary glands, gastrointestinal tract, subcutaneous abdominal fat) are managed according to routine techniques. In all cases close attention is necessary to preserve material for proteomic analysis.

With regard to abdominal fat tissue, the authors suggest skin punch biopsy or surgical subcutaneous fat biopsy rather than fine needle aspiration biopsy or needle biopsy with wider diameter, in order to obtain more material. Moreover it is advisable that fat tissue arrives fresh at the Laboratory, although some centers prefer it to be immediately immersed in the fixative.

In some centers thicker sections (5–10 μm) for CR are used in order to increase sensitivity in detection of amyloid deposits, but it should be said that automated stainers eliminate the problem (14).

### Standard histopathologic examination

The initial step in tissue amyloidosis diagnostics is morphologic identification or a suspicion of amyloid deposits in routine Hematoxylin-Eosin slides.

**FIGURE 1:**
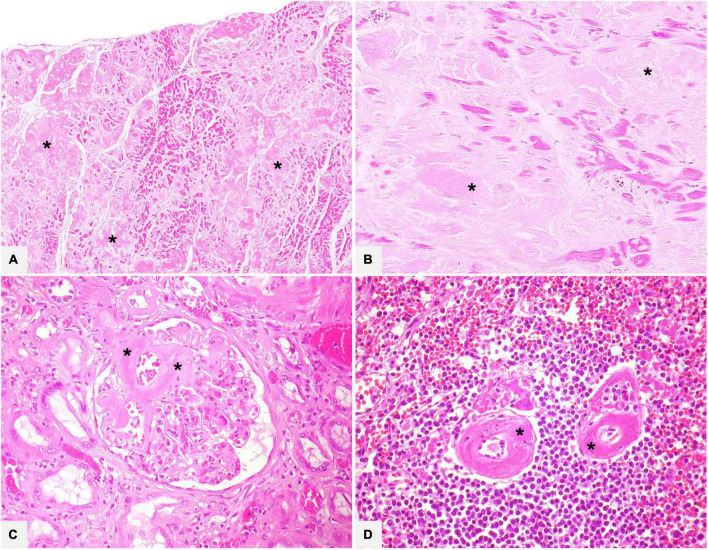
Various organ specimens (**A:** heart left atrium; **B:** heart left ventricle; **C:** kidney; **D:** spleen) in cases of systemic amyloidosis showing extracellular eosinophilic, amorphous, and homogeneous amyloid deposits (asterisks). Hematoxylin-Eosin: **(A)** 50×; **(B)** 200×; **(C,D)** 400×.

All amyloid deposits consist of fibrillary proteins with similar structure, which at histology appear as extracellular eosinophilic acellular, amorphous, and homogeneous material: this morphology is identical in each organ and in any form of amyloidosis and should principally be differentiated from collagen deposition, especially in the form of hyalin fibrosis, and from elastin (21) (Figure 1).

**FIGURE 2:**
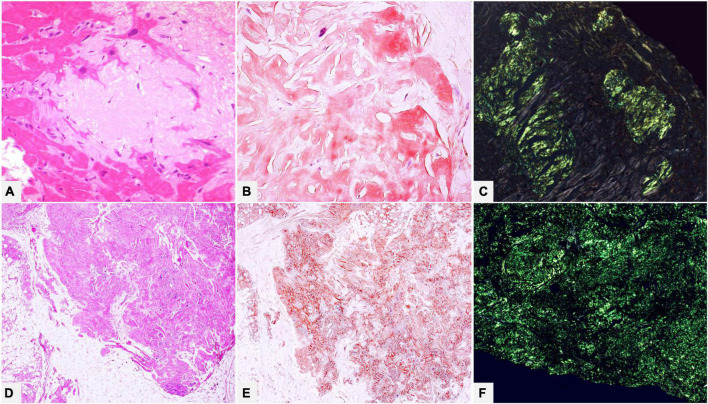
**(A–C)** Endomyocardial biopsy of a 52-year-old male patient with monoclonal gammopathy of undetermined significance: histology shows interstitial myocardial nodular amyloid deposits. **(D–F)** Patient of 15 years suffering from type I diabetes with insulin replacement therapy, who underwent surgical removal of abdominal fibro-lipomatous mass with diffuse interstitial nodular amyloid deposits. With Congo red, amyloid appears orange-red when viewed under transmitted-light microscope **(B,E)** and shows brilliant green birefringence under polarized light **(C,F)**. **(A)** Hematoxylin-Eosin 400×. **(B)** Congo red 400×. **(C)** Congo red under polarized light 200×. **(D)** Hematoxylin-Eosin 100×. **(E)** Congo red 100×. **(F)** Congo red under polarized light 200×.

The subsequent step is using CR stain, which displays a classic birefringence of amyloid fibrils when placed between two polarizers, thus confirming deposition. CR is the universal staining performed in pathology for amyloid assessment and the current gold standard for generic diagnosis of amyloidosis (Figure 2). When CR is negative in presence of a well-founded morphologic suspicion, the stain should be repeated in two or more sections in order to exclude technical problems. Including CR stain in protocols of major organ biopsies may be recommendable to reveal early, not yet morphologically evident, amyloid deposits or to identify limited deposits in clinically unsuspected cases, especially in organs such as kidneys where the clinical symptoms may not be very clear. It should, however, be stressed that a diagnosis of amyloidosis based only on CR birefringence in absence of histologic evidence should be proposed with caution (see below).

Congo red interpretation requires experience because dye results can vary considerably: high-quality microscopic optics, adequate observation conditions (strong light source, room darkness, quality of polarizers), and standardized staining protocols, both manual and automated, are required. Scant versus extensive deposits, pathologist’s experience, interobserver variability, tissue source (fat pad biopsy or aspirate vs. organ biopsy) are other factors which can substantially affect the results (22–25). The most frequent definitions of amyloid fibril birefringence are “typical green or apple-green birefringence” (Figure 2), but several papers express reservations about this terminology. In clinical practice, especially when the above-mentioned requisites are not observed, a mixture of colors, more commonly green, yellow, orange-red, blue-green, and whitish may be seen at microscope (7, 14, 26).

To cope with this diagnostic difficulty, which may not be confined to CR, the best approach is to carefully compare morphological findings, i.e., the deposits identified at histology, and birefringence tissue sites.

Types and characteristics of amyloid fibrils (such as full-length, thickness, truncation) can also influence intensity of birefringence, thus making it more difficult to diagnose some types of amyloidosis (for example, transthyretin amyloidosis) (27–29).

Other stains such as metachromatic dyes, Alcian blue (which binds to the ever-present glycosaminoglycans in fibrils) and Thioflavin T (or S), are not generally used in referral pathology laboratories, or may be used as additional staining.

**FIGURE 3:**
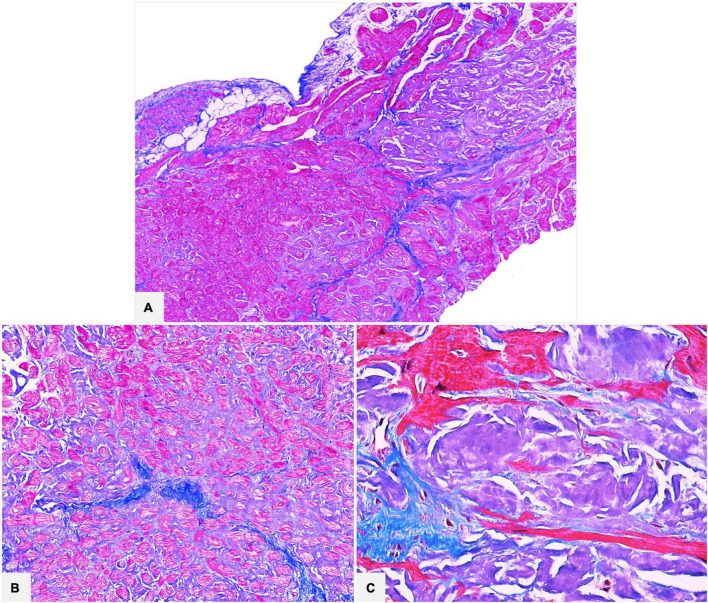
Cardiac specimens from native hearts of patients transplanted for ATTR. The myocardial interstitial pericellular and nodular amyloid deposits are stained bluish-gray with Azan Mallory trichrome and are clearly distinguishable from the brilliant blue collagen deposition (**A:** 50×; **B:** 100×; **C:** 400×).

**FIGURE 4:**
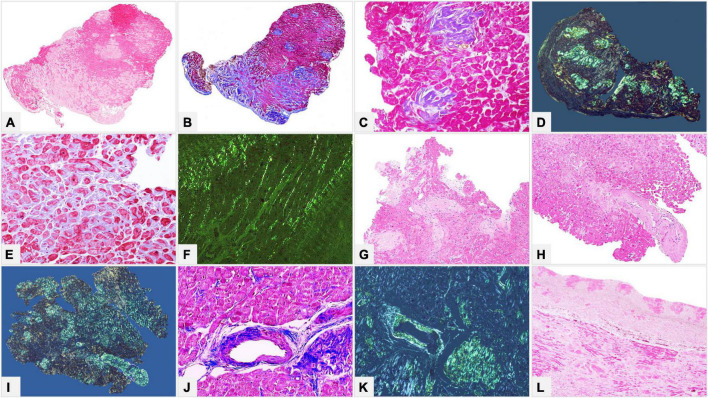
**(A–K)** Endomyocardial biopsies of patients with AL and ATTR. **(L)** Atrial sample of native heart of a patient transplanted for ATTR. **(A,B)** Myocardial interstitial mixed pericellular and nodular patterns diffusely distributed throughout the biopsy fragment. **(C,D)** Myocardial micronodular pattern, where amyloid aggregates replace the myocardium. **(E,F)** Myocardial interstitial pericellular pattern, made up of thin amyloid deposits around cardiomyocytes. **(G–K)** Amyloid deposits within mural arteries involving both intima and medial layers with circumferential **(G–I)** or focal **(J,K)** distribution. **(L)** Nodular amyloid deposits within subendocardial fibrous thickening. **(A)** Hematoxylin-Eosin 50×. **(B)** Azan Mallory trichrome 50×. **(C)** Azan Mallory trichrome 200×. **(D)** Congo red under polarized light 50×. **(E)** Azan Mallory trichrome 200×. **(F)** Congo red under polarized light 200×. **(G,H)** Hematoxylin-Eosin 100×. **(I)** Congo red under polarized light 50×. **(J)** Azan Mallory trichrome 200×. **(K)** Congo red under polarized light 100×. **(L)** Hematoxylin-Eosin 50×.

Azan Mallory trichrome stain shows amyloid as bluish-gray and helps to identify the deposits, to distinguish them from collagen and to evaluate the disease extent in bright field microscope (Figures 3, 4).

In our pathology center a consolidated quality control (QC) system ensures that CR and other staining protocols fall within quality specifications. Expert pathologists and technicians work together to regularly check the QC trends.

## Further information provided by histology evaluation

### General points

Although amyloidosis may be found in a localized form, it is most frequently a systemic disease, which involves numerous organs and tissues, more commonly heart, kidneys, nervous system, liver and gastrointestinal tract and, less commonly, lung, muscle, and soft tissue. Amyloidosis diagnosis may involve a general pathologist, but the disease is basically organ-specific with organ-related histopathological patterns, and ideally requires specialized pathologists (cardiopathologist, hematopathologists, nefropathologist, neuropathologist).

In addition to a definite diagnosis of organ involvement, histology evaluation of an organ biopsy can provide a wide spectrum of findings relating to disease etiology and comorbidities, as well as the pathobiology of deposition and acute and chronic organ damage.

### Focus on the heart

In the most common forms of amyloidosis in Western Countries, the heart is frequently involved: immunoglobulin light chain amyloidosis (AL) due to clonal plasma cell dyscrasia, and transthyretin amyloidosis (ATTR), due to anomalies of transthyretin (TTR), which includes the most frequent acquired wild-type form (ATTRwt), where protein misfolding is age-related, and the hereditary variant form (ATTRv) where transthyretin is mutated. These forms account for 98% of cases with significant cardiac disease, which is known to be the main determinant of adverse clinical outcomes (29–32). Cardiac involvement is also clinically critical in Apolipoprotein AI amyloidosis (AApoA1), while it is very rare in patients with reactive systemic amyloidosis (AA) where fibrils are composed of serum amyloid A protein (SAA) (33).

In our Center we performed various pathological studies of whole hearts, which showed the clinical value of assessing morphological variability of amyloid infiltration in terms of anatomical structures involved and different distribution patterns in cardiac walls or along the base-apex axis (34–36).

Histomorphology clearly shows that cardiac amyloidosis is both a myocardial and a vascular/microvascular disease, each with different deposition patterns.

There are two main myocardial interstitial patterns: pericellular and nodular/replacement. In the former, amyloid deposits are distributed around individual cardiomyocytes, vary in thickness and can involve areas of varying extent, thus producing a lace-like aspect; in the latter, nodular or micronodular amyloid aggregates, whether large or small, can distort the myocardial architecture or replace the myocardium. The two patterns are frequently mixed and deposit extent can be graded as mild-focal, moderate-multifocal and severe-diffuse (37) (Figures 4A–F). Deposit extent can also be morphometrically evaluated.

Amyloid deposits can be seen in vessels of various size, both arteries and veins, at epicardial and intramyocardial sites, and more extensively in mural vessels (37). Deposits may entirely or partially involve vessel circumference, be localized only in the intima or the medial layer or in the whole wall and cause various degrees of stenosis to the point of obstruction. Capillary networks too may be affected and show reduced density (Figures 4G–K).

Amyloid deposition also occurs in the subendocardium, usually as nodular aggregates associated or not with fibrosis, and in the epicardial tissue (34) (Figure 4L).

**FIGURE 5:**
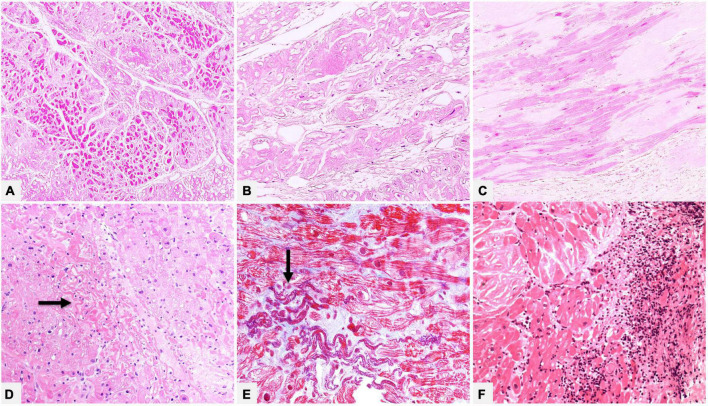
**(A–C)** Native heart of a 61 year-old male patient transplanted for ATTRwt. In the myocardial interstitium we can see amyloid deposits and morphologic chronic remodeling of cardiomyocytes, which show attenuation/atrophy (**A**, Hematoxylin-Eosin 100×), cytoplasmic vacuolization (**B**, Hematoxylin-Eosin 200×) or reactive hypertrophy (**C**, Hematoxylin-Eosin 200×). **(D,E)** Endomyocardial biopsy of a 55 year-old male patient with AL, where deposits prevalently involve vessel wall (not shown). There are foci of recent ischemic-like myocardial damage (thinned and wavy cardiomyocytes, hypereosinophilic and/or coagulated cytoplasm) (arrows). **(D)** Hematoxylin-Eosin 200×. **(E)** Azan Mallory trichrome 400×. Panel **(F)** shows an endomyocardial biopsy of a patient affected by ATTRv with extensive myocardial interstitial inflammatory infiltrates, mainly consisting of lymphocytes, associated with amyloid deposits and myocyte inflammatory damage (Hematoxylin-Eosin, 200×).

In amyloid cardiomyopathy histological examination can also give important information on myocardial injury. Most frequently chronic damage in a remodeling myocardium is found, characterized by various morphological alterations, such as attenuation/atrophy, vacuolization, or reactive hypertrophy of cardiomyocytes. In cases with significant microvascular involvement, it is occasionally possible to find myocyte ischemic-like damage at histology (Figure 5).

Although incidence of myocardial inflammation in cardiac amyloidosis is unknown, in our referral center, which handles many endomyocardial biopsies (EMBs) and numerous whole transplanted hearts of patients with amyloidosis, we had the opportunity to study myocardial inflammatory infiltrates associated to amyloid deposits, varying from simply lymphocytic or macrophagic-lymphocytic to giant cell granulomatous inflammation (Figure 5). Data are still scanty, but literature documented that amyloid may play a role in immune/autoimmune response (38). The issue of amyloidosis and inflammation is yet based on isolated and preliminary observations and research is needed to expand knowledge on pathogenetic mechanisms, possible role of inflammatory infiltrates on deposits, interactions with specific amyloidosis type and impact on disease progression and survival or on future therapeutic implication (39–41).

Finally, the presence of subendocardial and myocardial fibrosis should also be assessed to obtain information on overall morphologic alterations in the heart.

In conclusion histologic changes can provide substantial information on the multifactorial origin of cardiac damage and the complex pathophysiology of amyloidosis, whose varied clinical observations cannot be completely explained by the extracellular deposition of amyloid fibrils within the heart and the mechanical stress of deposits on myocytes. Direct cardiac toxicity of light chain precursor proteins in AL had been called into question, although the underlying mechanisms are not be clearly elucidated as well as a possible histologic expression of this type of damage (42, 43). Correlating systematically detailed morphologic patterns with clinical characteristics could provide further information to elucidate the spectrum of cardiac dysfunction, from altered ventricle relaxation to restrictive disease or to progressive systolic heart failure, and the mechanisms of myocardial ischemia or microvascular dysfunction-induced heart failure (44). It might also throw light on prognostic implications of cardiac disease burden and support the rapidly evolving field of therapeutic and drug efficacy research (45–48).

### Focus on the kidney

The kidney is the organ most commonly affected by amyloidosis; the associated renal dysfunction contributes to morbidity and mortality. Almost all the recognized amyloidogenic proteins can involve the kidney (49).

The prevalence of amyloidosis is estimated at 1.6% in native kidneys (50). The most common cause are AL (81%), AA (7%), and Leukocyte chemotactic factor-2 amyloidosis (ALECT2) (3%) (51, 52); by contrast with cardiac involvement, ATTR is an uncommon cause of renal disease (53).

Generally, all kidney biopsies are examined in light microscopy, immunofluorescence and electron microscopy. Under light microscopy, amyloid can involve any compartment of the kidney (glomeruli, tubules, interstitium, vessels) and distribution of deposits could vary with amyloid type (53, 54). Immunofluorescence is the most common method for AL diagnosis, where negativity for immunoglobulins and complement and positivity for one of the light chains is generally observed, with a sensitivity of 65–85% (55).

Several histological grading scores for renal amyloidosis have been proposed. One of the most used was suggested by Sen et al. in 2010 (1), based only on the glomerular pattern of injury. In 2017 Rubinstein et al. (56) proposed another score, validated in an AL cohort including glomerular, interstitial and vascular deposits, which was found to be predictive of end stage renal disease. More recently this score was also validated in an AA cohort (57).

## Typing amyloid

After histological diagnosis and description of organ-related morphologic findings, amyloid tissue typing, i.e., identification of fibril protein, is required as a guide to therapy, especially today when targeted therapeutic strategies are available for the two main types, AL and ATTR.

### Immunohistochemistry and immunofluorescence

The most common methods worldwide to determine fibril type are immunohistochemistry (IHC) on FFPE tissue, and immunofluorescence (IF) on fresh-frozen tissue, although they can be inconclusive or misleading, particularly outside centers of expertise (14, 16, 58, 59).

Immunofluorescence is the diagnostic gold standard in renal biopsies; for other organs IHC on FFPE sections is commonly used (14).

Optimal results with immunohistochemical stains are largely dependent on (58, 60, 61):

1. quality of antibodies;
2. experience of the pathologist;
3. standardized technical methods in local laboratories.

#### Commercially available antibodies

A number of antibodies and antibody panels are generally used in pathology diagnostics. Commercial antibodies for the most common fibril proteins (kappa and lambda immunoglobulin light chains, TTR, SAA) are usually employed, especially in non-specialized laboratories.

The main limitation of these antibodies is that they are produced against the native proteins, which have a regular length and conformation. Amyloid fibrillary proteins, however, are anomalous, often fragmented, and may present conformational and post-translational alterations, which can generate altered epitopes, or genetic mutations of amino acid sequence, which cause epitope loss and result in antibody-binding loss. In particular, the specificity of commercial antibodies against immunoglobulin light chains (IgLCs) is low, because they are produced against the constant regions and usually react with entire immunoglobulin; AL fibrils stem from mutations of the hypervariable region and require recognition of various antigenic specificities (62). Other factors such as non-specific signal interference due to tissue contamination from serum proteins, and cross-linking of proteins due to fixation in formalin can contribute to false-negatives or false-positives and to increased background. Lack of staining or, more frequently, multiple reaction of a single amyloid deposit with various antibodies can occur, especially with anti- TTR, anti-lambda and anti-kappa IgLCs, and even anti-AApoAI; these make it impossible to define amyloidosis type or can pose a problem for interpretation (63).

As the literature data on sensitivity and specificity of commercial antibodies vary considerably, it is really difficult to estimate their true accuracy in identifying fibril type in local laboratory routine practice (59, 64–67): special attention is required for IgLC staining low sensitivity and concomitant false-positive staining for TTR (67).

#### Amyloid-type specific antibodies

For these reasons, since early reports of unreliability of antibodies against proteins of origin (68, 69), some laboratories specializing in amyloidosis diagnostics and research have developed their own amyloid type-specific monoclonal and polyclonal antibodies to determine the amyloidogenic protein (70).

The most complete data in the literature is the work of Linke and colleagues who, over a number of years, developed a set of specific antibodies using a large number of tissues with chemically or immunochemically typed amyloids as prototypes, i.e., antibodies directed not against the precursor, but against the purified fibril protein (70). Linke et al. verified antibody performance by serial controls on a large number of prototype amyloids at their own and in other institutes, and also by mass spectrometry, so achieving high diagnostic accuracy on FFPE tissue with 97.9% sensitivity and 99.3% specificity (71). Using a reduced kit (anti-AA, anti-lambda and anti-kappa IgLCs, anti-TTR) for confirmation of a supposed amyloid, correct typing of these most common forms decreases to 90% (61) because IHC can find only the targeted amyloid types.

Similar excellent results with these antibodies, now commercially available in the form of kits for various purposes, are also reported by Lassner and Schonland (59, 71).

The use of specific and standardized antibodies considerably increases sensitivity and specificity of the IHC method, and allows correct fibril typing in a greater number of cases (72), although immunostaining of amyloid deposits by more than one antibody is not fully resolved with these antibodies (63, 73).

Despite these advantages it should be mentioned that it is difficult to use non-validated and non-commercialized specific antibodies in certified laboratories.

**FIGURE 6:**
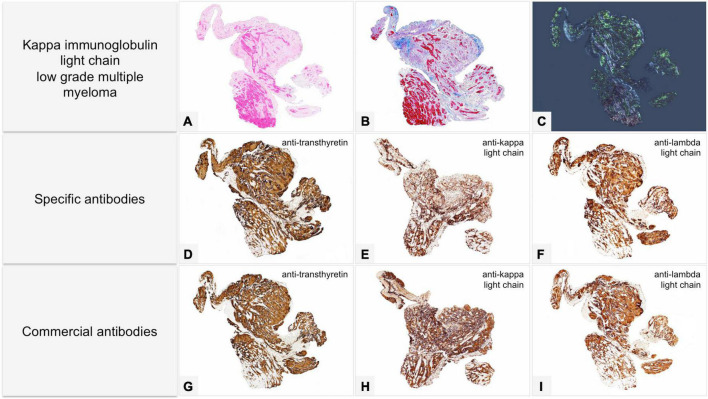
Male of 81 years affected by kappa light chain low grade multiple myeloma, with score 2 cardiac uptake on scintigraphy with bone tracers. Endomyocardial biopsy was performed for suspected cardiac amyloidosis and showed slight to moderate myocardial interstitial and subendocardial amyloid deposits. Proteomic analysis with mass spectrometry in tandem mode was positive for TTR proteotypic peptides. IHC failed to type amyloid with both commercial and specific antibodies. Immunolabeling was strong and diffuse with all antibodies with no significant differences in intensity and distribution. **(A–C)** Histology of a biopsy fragment showing subendocardial and myocardial eosinophilic amorphous deposits with Hematoxylin-Eosin (**A:** 50×), bluish-gray deposits with Azan Mallory trichrome (**B:** 50×) and green birefringent deposits with Congo red under polarized light (**C:** 50×). Specific antibodies (amY-kit reduced PeloBiotech). **(D)** Anti-ATTR-TIE (50×). **(E)** Anti-kappa-KRA/KUN (50×). **(F)** Anti-lambda-UTI/LAT (50×). Commercial antibodies against native proteins. **(G)** Anti-ATTR AbCam, clone EP2929Y (50×). **(H)** Anti-kappa Roche-Ventana (50×). **(I)** Anti-lambda Roche-Ventana (50×).

**FIGURE 7:**
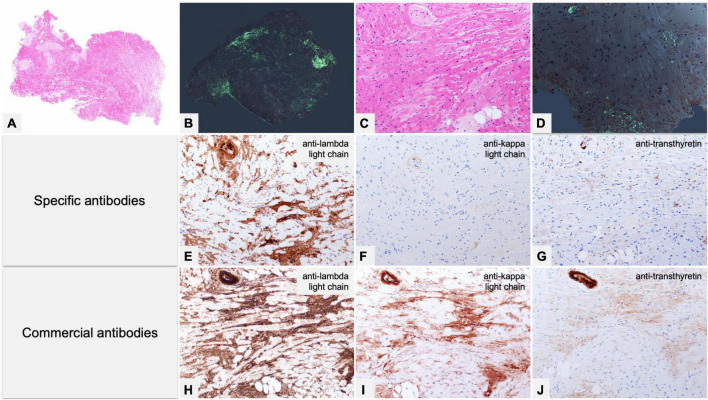
Endomyocardial biopsy of a 55-year-old male patient performed for clinically unexplained cardiopathy/cardiomyopathy. **(A–D)** Histology shows amyloid deposits within vessels and myocardial interstitium, clearly evident with Congo red birefringence **(B,D)**. **(E–G)** Immunohistochemistry with specific antibodies was suggestive of AL, as it showed strong immunostaining for lambda light chain in vessel and myocardial deposits, and substantial negativity for kappa light chain and TTR. **(H–J)** Immunohistochemistry with commercial antibodies was inconclusive: all three antibodies showed strong immunostaining of the artery, although myocardial interstitial amyloid deposits were particularly strong only with anti-lambda light chain, partially due to background. Fibril protein typing with mass spectrometry in tandem mode was positive for lambda light chain proteotypic peptides. Subsequent hematological study led to diagnosis of lambda light chain monoclonal gammopathy of undetermined significance and related AL principally localized in the heart. **(A)** Hematoxylin-Eosin 50×. **(B)** Congo red under polarized light, 50×. **(C)** Hematoxylin-Eosin 200×. **(D)** Congo red under polarized light, 200×. Specific antibodies (amY-kit reduced PeloBiotech): **(E)** Anti-lambda-UTI/LAT (200×). **(F)** Anti-kappa-KRA/KUN (200×). **(G)** Anti-ATTR-TIE (200×). Commercial antibodies against native proteins. **(H)** Anti-lambda Roche-Ventana (200×). **(I)** Anti-kappa Roche-Ventana (200×). **(J)** Anti-ATTR AbCam clone EP2929Y (200×).

**FIGURE 8:**
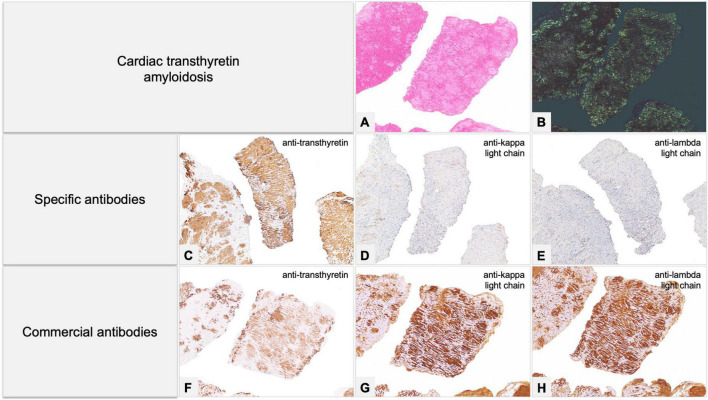
Endomyocardial biopsy of a 68-year-old male patient, performed for suspected cardiac amyloidosis in absence of hematological disease. **(A,B)** Histology shows extensive amyloid deposits involving myocardial interstitium, with a prevalent nodular pattern, and the vessels. **(C–E)** Immunohistochemistry with specific antibodies clearly favored an ATTR form, as it showed diffuse homogeneous immunostaining for TTR **(C)** and negativity for anti-kappa **(D)** and anti-lambda **(E)** immunoglobulin light chains. The result was confirmed by proteomics with mass spectrometry in tandem mode, which was positive for TTR proteotypic peptides. **(F–H)** Immunohistochemistry with commercial antibodies, however, showed weak immunostaining for TTR **(F)** and strong, diffuse positivity using anti-kappa **(G)** and anti-lambda **(H)** light chains. Genotyping allowed diagnosis of ATTRwt. **(A)** Hematoxylin-Eosin 50×. **(B)** Congo red under polarized light, 50×. Specific antibodies (amY-kit reduced PeloBiotech): **(C)** Anti-ATTR-TIE (50×). **(D)** Anti-kappa-KRA/KUN (50×). **(E)** Anti-lambda-UTI/LAT (50×). Commercial antibodies against native proteins. **(F)** Anti-ATTR AbCam clone EP2929Y (50×). **(G)** Anti-kappa Roche-Ventana (50×). **(H)** Anti-lambda Roche-Ventana (50×).

Exemplary clinical cases are shown in Figures 6–8.

#### Expertise of the pathologist and the laboratory

As for CR, interpretation of immunostainings requires a pathologist with extensive experience, who should also be familiar with different immunolabeling patterns (69).

Potential pitfalls include not only discerning non-specific background but also amyloid specific staining, which can vary in terms of distribution both over all deposits and within single deposits. Immunolabeling can appear uniform or non-homogeneous, equally distributed or spotty and widespread or limited; reactivity intensity may be strong or weak.

A caution approach is needed for inconsistent and variable immunolabelling patterns; strong, uniform and coherent immunostaining can usually be considered diagnostic, as long as the results are correlated with clinical data (69).

Pathology laboratory technicians should be able to perform all technical steps to guarantee the best performance of antibodies and methodology should be standardized and reproducible. The aims of the IHC standardization process are to optimize specific versus background staining and to select the intensity of immunolabeling using positive and negative controls.

Today’s automated platforms are an additional guarantee for adequate standardization as compared to manual platforms (60).

### Proteomics

Mass spectrometry (MS)-based proteomic analysis of amyloid deposits has been shown to identify fibril subtype with a high degree of accuracy, and is therefore considered the gold standard technique in amyloidosis typing (74). Vrana et al. first developed the procedure for FFPE organ biopsy specimens (75), and then for unfixed fat aspirate specimens (76).

The procedure uses the shotgun proteomics approach to analyze specific areas of CR positive tissue viewed under polarized light. The areas are first resected by laser microdissection (LMD), then collected in a microvial, solubilized and further processed to obtain a peptide solution mixture, which is in turn analyzed by nano-flow liquid chromatography (nf-LC) coupled to high-resolution and high-accuracy mass spectrometry in tandem mode (hr-MS/MS). The collected spectra are checked through database matching software, such as Sequest (77), Tandem (78), or Mascot (79), in order to identify proteins.

The software usually uses tryptic peptide databases containing sequences for the Swiss-Prot human canonical proteome, that is sufficient to identify virtually all amyloid proteins in specimens. But in cases of hereditary amyloidosis, where mutated proteins are present, special databases have been developed to identify mutated peptide sequences, although these are only available in a few specialized centers worldwide (74, 76).

The shotgun proteomics approach usually detects amyloidogenic fibril proteins in samples, together with many additional proteins (17). As chaperone proteins involved in the amyloidogenesis process have commonly been found (80), independently of the specific fibril forming protein which changes in various amyloidosis types (17), their presence has been proposed as an amyloid molecular signature.

In the diagnostic evaluation of bioptic specimens, the general strategy would be: when the signature is detected, an amyloid type is identified by correlating patient clinical factors with the most abundant amyloidogenic protein consistently found in a series of repeated proteomics analyses.

However, when a CR-positive sample contains the biochemical signature of amyloidosis but not a known amyloid type, the proteome can be further scrutinized for potential novel amyloid fibril forming proteins. Using this approach, various previously unknown fibril proteins, such as leukocyte chemotactic factor-2, apolipoprotein A4, apolipoprotein C2, liraglutide, and epidermal growth factor containing fibulin-like extracellular matrix protein, have been identified as novel amyloid types with very different clinical presentations and outcomes (17).

After scrutinizing more than 16,000 cases, Vrana et al. (76) were able to indicate a universal amyloid signature composed of Apolipoprotein E (ApoE), Serum amyloid P component (SAP), and Apolipoprotein A-IV (ApoA-IV), since these were invariably present in their pathological deposits. They went on to assert that, when found together, these three proteins constitute a biochemical signature and support the general diagnosis of amyloidosis, even independently of CR staining results.

Recently, however, other authors have proposed slightly different amyloid signatures: Misra et al. (81) indicated ApoE, SAP, and glycosaminoglycans; Benson et al. (1) proposed ApoE, SAP and heparan sulfate proteoglycan; Schumann et al. (82), using a new proteomics approach based on MALDI imaging, have found an even more elaborate signature composed of ApoE, SAP, Apolipoprotein A-1, Vitronectin, and SAA.

Although the shotgun proteomics approach is rightly considered the “gold standard” method in the diagnosis of amyloidosis type, in practice technical complexities, sample recovery issues, processing, microdissection, data analysis, and the availability of expensive instrumentation and plus multidisciplinary professional team restrict this approach to only a few reference centers throughout the world (54, 83).

Hence, in an attempt to implement MS-based amyloid typing in our center, we developed a targeted proteomic approach based on standard liquid chromatography and mass spectrometry (LC-MS)/MS instrumentation by limiting the number of proteins sought in both FFPE biopsies and subcutaneous fat aspirated (SFA) samples (84). In addition to proteotypic transitions of fibril-forming proteins, we included Cardiac Actin for EMBs and Fatty Acid Binding Protein-4 for SFA specimens. Identifying these last tissue specific proteins served not only as a tissue marker, but also to define relative cut-off values of fibril proteins in positive samples and to rule out false positivity due to monomeric forms circulating physiologically in the human body and accidentally included in bioptic specimens (84). We have recently started to include the amyloid signature in our method involving the presence of three chaperone proteins (ApoE, SAP, ApoA-IV): as these are, however, physiologically present in the patients’ bloodstream, their presence is not necessarily an unequivocal marker of amyloidosis, but could merely indicate blood inclusions in bioptic specimens.

Our method can be performed on standard 2–5 um thick sections of FFPE tissues (or small chunks of SFA specimens) positively stained with CR. Without using LMD the sections are transferred whole from the glass slits to an eppendorf. The tissue is then solubilized, proteins are extracted, denatured and digested by trypsin, using a commercial kit of reagents for shotgun proteomics (Easypep mini, Thermo-fisher, Waltham, MA, USA). The resulting peptide mixture is analyzed by a standard LC approach, on 2 × 150 mm, 1.8 um particle size peptide specific columns, coupled with triple quadruple tandem mass detection in multiple-reaction-monitoring (MRM) mode. Peptide specific MRM transitions for proteotypic peptides are obtained *via* an open access database: the SRMAtlas.^1^ We developed the method with two different systems, with similar successful results: Nexera-2 UPLC (Shimadzu, Kyoto, Japan) coupled with an API5500 mass spectrometer (Sciex, Toronto, Canada); and 1295C UPLC (Agilent, Santa Clara, CA, USA) coupled with a 6495C mass spectrometer (Agilent, Santa Clara, CA, USA).

In our targeted approach, the presence of chromatographic peaks for specific mass transitions in ion extraction chromatograms is used to verify the presence of proteotypic peptides. At least three positive proteotypic peptides is indicative of the presence of a target protein in a sample.

However, since the SRMAtlas database contains only sequences for SwissProt canonical human proteome sequences and does not contemplate amino acid substitutions, less conserved canonical proteotypic peptide signals can be used to presume identification in expected hereditary forms, despite reduced identification confidence.

In our Center, for the last year, amyloid typing protocol has included a first IHC screening level, using commercial antibodies and two sets of specific ones (one marketed by Pelobiotech GmbH-Germania and the other developed by the University of Uppsala not currently marketed) and, in inconclusive or dubious cases, LC-MS/MS as a second tier test in order to pinpoint any false IHC results (17). At the end of the typing process, data from the two levels are compared and matched with clinical data. Preliminary results of this sequential approach show that using specific rather than commercial antibodies substantially increases correct amyloid typing by as much as 70% of cases, so reducing the need to proceed to proteomics.

In perspective, Mass Spectrometry Imaging (MSI), a technique where an ion or a laser beam is raster scanned over the tissue surface to vaporize it into molecules that are then immediately transferred to the mass spectrometer, has the potential to go beyond shotgun proteomics in amyloid typing (85, 86) thanks to its ability to preserve spatial distribution of proteins in tissues and therefore allow direct observation of the molecular composition of amyloid deposits (87). In addition, the MSI approach can permit direct localization of other types of biomolecule, such as lipids (88) or metabolites in bioptic specimens (89), and could provide new insights into the process of fibrillar protein aggregation, today still largely misunderstood (90).

Nanometric spatial resolution, however, is still impossible with MSI at its present level of development and technical improvements are needed for its successful application in clinical pathology (82).

### The question of immunoelectron microscopy

Immunoelectron microscopy (IEM) combines IHC and electron microscopy (EM). This technique is based on extreme microscopic magnification that allows amyloid fibrils to be visualized. With colloidal gold-labeled specific antibodies, it is possible to see whether the antibodies bind specifically or un-specifically to the amyloid fibrils, so overcoming the low specificity of standard IHC. IEM has been successfully established at some amyloidosis centers (91), but its use requires great caution by both clinicians and pathologists. To obtain optimal results, the sample cannot be fixed in 2,5% Glutaraldehyde (as for transmission EM), but in 0.5% Karnovsky’s solution (0.5% glutaraldehyde, 2% paraformaldehyde in 0.2 M cacodylate buffer, pH 7.3), as described by Arbustini et al. (92).

Although the availability of IEM is limited, in experienced centers it seems to obtain excellent results. In a single-center study of 423 cases of systemic amyloidosis, IEM identified the amyloid type in over 99% of cases (93). A recent study compared IEM and MS for amyloid subtyping: IEM defined amyloid type in 91.6% of cases and MS 88.8%, over 106 biopsies from different organs; the authors also support the combined use of both methods to increase the sensitivity of defined amyloid type and mention the important issue of tissue amount in a diagnostic routine, indicating that MS requires a very small amount (0.1 mm^2^) and IEM a little more tissue (1 mm^2^) (94).

As usual, the perfect method does not exist and few papers in the literature give a comparison between IEM and MS (95).

Potentially both methods can be performed on FFPE material, although it is particularly challenging for IEM outside expert centers which use this technique routinely. Very few laboratories are able to use the paraffin recovery technique, even fewer from a stained slide (96), partly due to the present reduction of clinical indications for EM in other pathologies. Like IHC, IEM uses antibodies, so it can identify only the fibrils present in the antibody panel, while MS can potentially identify all proteins. MS requires as little as two working days laboratory time, whereas IEM needs a minimum of 7. Both techniques are expensive and depend on equipment and staff. It must be emphasized that, as clinicians request biopsies less frequently, it is very difficult to find technicians and pathologists trained in EM, so many hospitals are abandoning its use.

### Summary

We believe that the targeted sequential IHC with specific antibodies/LC-MS approach is most probably the gold standard for amyloid typing for many reasons (16, 61, 72):

1. Immunohistochemistry is a simple, quick, inexpensive method, available in most pathology laboratories and can obtain excellent results when using “good” antibodies in expert Centers;

LC/MS is the most sensitive method, able to provide accurate protein information and, using an extended database, to identify mutations and, potentially, novel forms.

In not so rare cases with a real coexistence of a mixed protein population, immunohistochemical data are essential to proteomics, which can have difficulty in recognizing whether proteins stem from amyloid. This may occur in ATTR cases where circulating kappa IgLCs can contaminate TTR fibrils, or in AL cases where kappa or lambda IgLCs can produce a nest effect and attract circulating wild-type TTR, or especially when a monoclonal gammopathy of undetermined significance (MGUS) coexists with ATTR, which can occur in 10–49% of patients (97).

Finally, when a diagnosis of ATTR is reached, genotyping is mandatory.

## Histologic evaluation: When and where

Histologic identification of amyloid deposits on tissue specimens is the most sensitive method for definitive amyloidosis diagnosis, and a sequential approach with IHC and proteomics the most sensitive method for amyloid fibril typing and diagnosis of amyloidosis type. Thus in such a clinically complex disease having tissue available is of great importance.

When amyloidosis is suspected, tissue biopsy is always required except for cases of cardiac ATTR, which may be diagnosed by non-invasive methods when the following stringent criteria are met (98): patient with signs and symptoms, electrocardiography, echocardiography, or cardiac magnetic resonance suggestive of cardiac amyloidosis, Perugini score 2 or 3 (99) cardiac uptake on scintigraphy with bone tracers, and absence of monoclonal proteins examined with serum free light chain quantification and serum and urine immunofixation (21, 100). In the absence of these criteria, histological confirmation of diagnosis and/or typing is required (21, 100).

In the last decade the evolution of non-invasive diagnostic methods, specifically cardiac scintigraphy with bone tracers, has gradually changed the diagnostic approach to and clinical management of cardiac ATTR. So, although from the 1990s until the early 2000s EMB was frequently used in referral centers, today it is less common (41, 101).

It should be remembered that a bone scintigraphy scan alone is not enough to distinguish ATTR from AL cardiomyopathy without also testing for IgLCs (102).

In AL histological confirmation is mandatory as in all neoplastic diseases, where complex therapeutic protocols with many side effects are necessary (103).

### Biopsy sites

**FIGURE 9:**
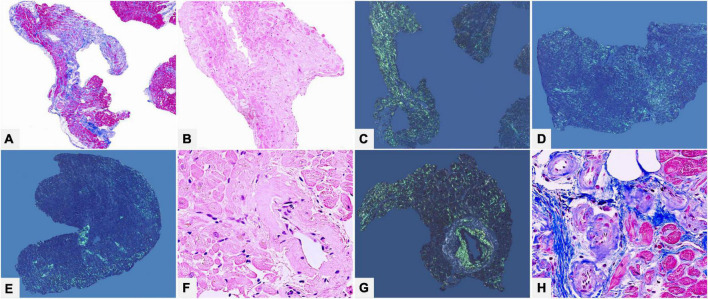
Male of 62 years affected by clinically not well defined cardiopathy with hypertrophic phenotype. Thorax computed tomography showed mediastinal lympho-adenomegaly and a pulmonary picture suggestive of grade 2 sarcoidosis. Endomyocardial biopsy identified cardiac amyloidosis with diffuse myocardial interstitial **(A–D)** and vascular **(E–H)** deposits. Immunohistochemistry typing (not shown) suggested AL, most probably related to lambda light chain, later confirmed by proteomics. Subsequent hematological tests confirmed the disease and a bone marrow biopsy defined the underlying plasma cell dyscrasia: low grade multiple myeloma. The final diagnosis was systemic lambda light chain amyloidosis with lymph node and cardiac involvement in clinical onset stage I. **(A)** Azan Mallory trichrome 50×. **(B)** Hematoxylin-Eosin 100×. **(C,D)** Congo red under polarized light, 50×. **(E)** Congo red under polarized light, 50×. **(F)** Hematoxylin-Eosin 400×. **(G)** Congo red under polarized light, 100×. **(H)** Azan Mallory trichrome 400×.

The most sensitive amyloidosis diagnostic method is a biopsy of a clinically involved organ, such as kidney (sensitivity: 99%) or heart (sensitivity: 100%): organ biopsy is the first choice in localized amyloidosis forms or when other diseases must be excluded in clinically unclear cases (Figure 9).

Although organ biopsy is probably the most used, but for some authors overused (104), in screening of systemic amyloidosis a surrogate biopsy site may be useful, to avoid the invasiveness of organ biopsy and possible additional bleeding diathesis in patients (98, 104, 105).

The choice of the correct site is crucial because tissue sensitivity in amyloid detection depends on type of suspected amyloidosis, as AL deposits are more likely to be identified than ATTR ones.

The most used alternative sites are: subcutaneous abdominal fat, gastrointestinal tract (usually rectal biopsy) and minor salivary gland biopsy.

**FIGURE 10:**
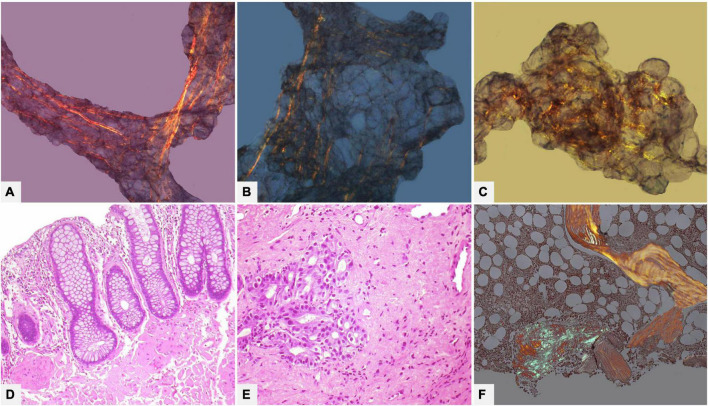
Various tissue and organ biopsies of various patients with AL systemic amyloidosis. **(A–C)** (100×): Subcutaneous abdominal fat biopsy showing ambiguous birefringence, yellow and focally green, with Congo red under polarized light. **(D)** Rectal biopsy with mucosal and submucosal amyloid deposits (Hematoxylin-Eosin 200×). **(E)** Gastric biopsy with extensive submucosal amyloid deposits (Hematoxylin-Eosin 200×). **(F)** Bone marrow biopsy with interstitial nodular amyloid deposit with congo red under polarized light (50×).

Abdominal fat tissue aspirate or biopsy is the most used for screening, especially in AL forms where sensitivity is quite high, ranging from 70 to 90%; fat tissue is inadvisable when ATTR is suspected because sensitivity is only 67% in ATTRv and as low as 14% in ATTRwt (106–110). Apart from the small amount, the main problem of abdominal fat tissue is the presence of fibrous strands, where CR birefringence is yellow or yellowish-green, making staining interpretation ambiguous and difficult (Figure 10).

For labial salivary gland biopsy high sensitivity (from 81 to 89%) is reported in AL as well as in familial amyloid polyneuropathy (91% of cases) (106, 111–113).

The sensitivity of gastrointestinal biopsies in general ranges from 70 to 90% (Figure 10). In particular, with rectal biopsy, sensitivity is high for AL (85%) (Figure 10) and ATTRv (81%) and low for ATTRwt (50%) (106, 107, 114).

Bone marrow biopsy deserves separate discussion. In the context of a clinical syndrome compatible with AL and of light chain abnormal findings, the screening work-up includes an iliac crest bone marrow biopsy with the main aim of checking for possible clonal plasma cell disorders. In such cases the biopsy protocol also includes Congo red stain to screen possible amyloid deposits, with a diagnostic yield of 50–60% of AL cases (but remember only of 30–40% of ATTR cases!). If deposits are found (Figure 10) the nature of localized or systemic amyloidosis should be determined: the characteristics of clinical syndrome and the pattern of organ involvement are the main guides to deciding whether proceed with a biopsy of extra-bone marrow tissue, keeping in mind that the probability of Congo red positive bone marrow developing systemic amyloidosis is very low (106, 115–117).

Another separate discussion is required for transverse carpal ligament biopsies obtained during carpal tunnel syndrome (CTS) surgery. Although CTS is a recognized red flag for ATTR cardiac amyloidosis, which can precede diagnosis by 5–9 years (118), there are very few studies that use the carpal ligament biopsy for screening purposes. One of these studies found 10.2% of amyloid positive specimens, but just 2% of these were ATTRv and a further 2% had cardiac amyloidosis (119); different results were reported in a Japanese cohort (120). Nowadays the histological diagnosis of carpal ligament biopsies is performed by only a few centers, partly because the presence of recent and older collagen fibrous tissue in these samples makes the analysis challenging (121).

## Conclusion

In amyloidosis, pathology study offers much key information in both diagnostics and research. Notably histology has been and continues to be an essential tool for reaching a definite diagnosis, excluding other diseases, classifying systemic and localized forms, describing organ involvement patterns and disease burden.

Screening or organ biopsies have been crucial to increasing knowledge of the disease since the 1990s and, today, are still essential for fibril protein typing and meeting the increasing clinical need for early diagnosis and treatment within a multidisciplinary collaboration scenario.

## Author contributions

MR and OL contributed to the conception and design of the study and wrote most of the manuscript. MC and BF wrote sections of the manuscript. SL critically revised the manuscript. All authors contributed to manuscript revision and read and approved the submitted version.

## Footnotes

1
www.srmatlas.org
